# New design of high-power in-motion inductive charger for low power pulsation

**DOI:** 10.1038/s41598-023-44949-z

**Published:** 2023-10-19

**Authors:** Ahmed A. Shaier, Ahmed A. S. Mohamed, Hamid Metwally, Sameh I. Selem

**Affiliations:** 1 Electrical Power and Machines Department, Faculty of Engineering, Zagazig University, Zagazig, Egypt; 2 Eaton Research Labs, Eaton Corporate, Golden, CO USA

**Keywords:** Engineering, Electrical and electronic engineering

## Abstract

The magnetic coupler is the most vital component for charging EV wirelessly. Through it, the output power can be transported from the transmitter to the receiver by means of electromagnetic fields. Therefore, this manuscript presents a proposed design of a magnetic coupler in the form of Double-D (DD) on both sides, which is suitable for in-motion inductive charging. This charger is capable of transferring power of 200-kW through an airgap of 250 mm with an efficiency of 91.88% and an operating frequency of 85 kHz. Computational modeling is conducted to obtain the magnetic coupler and the compensation parameters of the proposed system. The appropriate dimensions of the coils, magnetic and metallic shielding are obtained by using the finite element model (FEM). The effect of misalignments on the self and mutual inductances of the two coils (*L*_*p*_,* L*_*s*_, *M*), the output power (*P*_*o*_), and the transmission efficiency (*η*) is studied in case of one and two coils at transmitter side. The output power in the distance between the two transmitter coils (*d*) is improved by controlling the operating frequency, adding magnetizable concrete (MC), or both together. These techniques have proven effectiveness in improving the output power by 45.15% for small *d* and 72.51% for large *d*. In addition, the efficiency improved by 15.95% for small *d* and 60.76% for large *d*. Moreover, these improvement cases were compared in terms of size, weight and cost for a 100-m driving track.

## Introduction

Wireless charging for electric vehicles (EVs) is a promising alternative to plug-in charging due to its features including, safety, reliability, efficiency and automation. There are three charging manners for electric cars: stationary (static), quasi-dynamic, and in-motion (dynamic) charging^1^. In contrast to stationary inductive power transfer (SIPT), dynamic inductive power transfer (DIPT) can charge the battery of electric cars while driving during long trips on highways without the need to wait or stop to finish the charging process. In-motion charging technology has several advantages including, the ability to overcome range anxiety, reduce battery size, increase driving range on highways, and the possibility of designing and developing self-driving and controlling electric cars, which saves time and effort during the driving process^2^.

**Figure 1:**
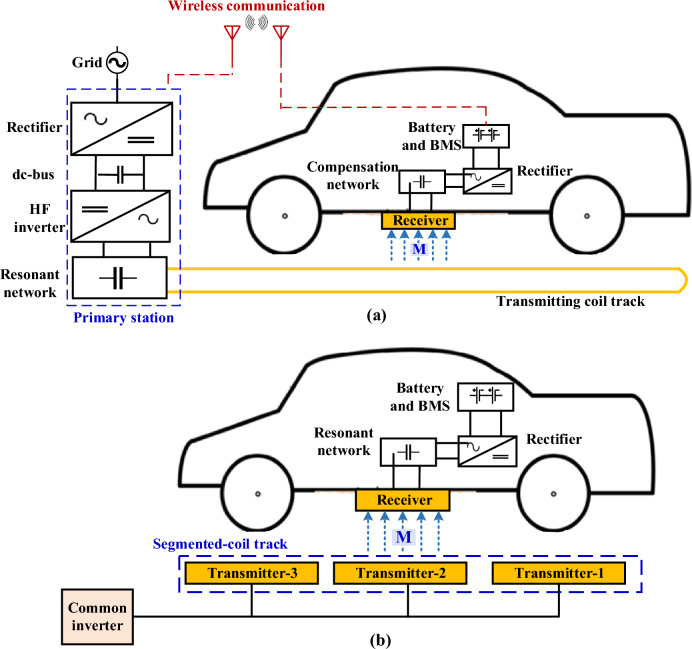
Various configurations of in-motion inductive transmitter, (**a**) single long coil track, and (**b**) separated coil track.

As in SIPT, there are two pads in in-motion charging, one of which is suspended at the bottom of the vehicle, and the other is buried under the surface of the ground. Those that are located on the ground side can be found in two configurations. The first is that the charging path consists of a single long coil as pictured in Fig. 1a, and the second is that there are several separate coils connected together in series or in parallel to form the charging track (separated coil array) as depicted in Fig. 1b^3^. There are two arrangements for feeding the separated coil path, one of which uses a common high frequency (HF) inverter to feed all separated pads, while the other one has an independent HF inverter for each pad through which the feeding is supplied^2^.

In order to achieve a high-power transfer with maximum efficiency during in-motion charging, most researchers have conducted several studies, especially on the segmented transmitter coil configuration. The charging path in this configuration consists of several separate coils similar to those used in SIPT, such as rectangular, circular, double D (DD), double D quadrature (DDQ), bipolar, tripolar, DQD, etc. The authors in^4^ propose a multi-objective optimization method to design a DD-shaped transmitting coil suitable for inductive charging systems. Several algorithms were used to obtain the best design by trying to obtain a good coupling coefficient while minimizing leakage electromagnetic fields. This study was conducted to comply with power Level 2 recommended by the standard SAE 2954. In^5^, power has been transferred with an efficiency of 85% by proposing a dynamic charging system. This system selects the length of the transmission coil based on the transmission efficiency, the output power, the energy consumed per km and the power loss. The authors in^6^ propose a transmitter composed of separate coils, each of which is a DD configuration. The mutual inductance and the appropriate dimensions of the coils were calculated. A power transmission of 2.5-kW with a dc-dc transmission efficiency of 85% was achieved by researchers in^7^. Where a mixture was made between the rectangular and the DD coils to form the transmission path. In^8^, a 5-kW power transmission in-motion charging system was proposed with an efficiency of 92.5% at an operating frequency of 20 kHz. In^9^, a dynamic charging system with LCC compensation technology was used to transmit 2.34-kW power with an efficiency of 91.3%. The maximum power pulsation is ± 2.9% of the average output power.

In^10,11^ the behavior of different compensation networks used in in-motion charging has been studied. In^12^, an in-motion charging system with LCL-LCL compensation network was proposed and a power of 7.36-kW was achieved with an efficiency of 95.8%. In^13^, a T-type compensation was used to transfer power through a dynamic charging system, where an 85% transfer efficiency was achieved. In addition to maintaining the power fluctuation by less than 6% of the average output power value. In^14^, an in-motion charging system was proposed that studies the use of LCC compensation at the transmit side, with series compensation and LCC compensation at the receiver side. It was achieved that the transmission efficiency of this system is improved by 7%. The researchers in^15^ proposed an in-motion charging system that works to reduce the fluctuations occur in the power while charging the EV battery. Several rectangular coils were used in the transmitter side, while one coil was used in the receiver side. The transmitter coils are arranged very close to each other in order to reduce fluctuations in the magnetic field along the charging path. By means of this design, a power transmission of 1.4-kw was achieved, with an dc–dc efficiency of 89.78%, and power fluctuations of ± 7.5%.

In^16^, the authors inserted bipolar coils with unipolar transmitter coils to form a dynamic charging system. This combination was made on the transmitter side in order to eliminate the self-coupling that arises between the unipolar coils, and it also helps to obtain a stable coupling coefficient when the receiver coil moves along the charging path. The required power was transmitted with an efficiency of 90%, and a power fluctuation of ± 2.5%. In^17^, a dynamic charging system consisting of three-square pads was built on the receiver side while three rectangular pads were installed on the transmitter side. An output power of 30-kW was obtained with an efficiency of 88%. In^18^, the authors designed a three-phase dynamic charging system with the transmitter and receiver sides. This design is suitable for charging heavy-duty EVs, in addition to eliminating power fluctuations. A simulation of a 200-kW/m dynamic charging system was carried out to emphasize the principle of eliminating power fluctuation. Moreover, a prototype that transmits 52-kW and achieves the least fluctuation in power is established.

In^19^, a design for an in-motion charging system was created that transfers power of 200-kW with an efficiency of up to 92% and using of an LCC-S compensation network at frequency of 85 kHz. This design was studied when a single coil is used for both the transmitter and the receiver each on a DD configuration. The thermal effect of the pavement on the system was studied, in addition to the safety degree achieved by this system, where it was found that it did not exceed the standard limits of electromagnetic fields. In^20^, an analytical study was carried out on the sensitivity of an in-motion charging system to the occurrence of lateral misalignments. This system transmits power of 200-kW by using bipolar coils at both transmitter and receiver and in the presence of compensation networks such as LCC-S and LCC-P at frequency of 85 kHz.

The contributions in this work are:

- Design an in-motion charging system capable of transmitting high power of 200-kW with high efficiency 91.88% at a relatively large air gap distance (250 mm) according to the Society of Automotive Engineers (SAE J2954/3) standard version 3. The segmented coil path that is divided into multiple transmitters is considered.
- The transmitter and receiver coils are designed in the form of a Double D (DD) based on 3D finite-element analysis (FEA), in addition to the magnetic and metal shielding are designed. An appropriate LCC-S compensation network has also been designed.
- Study the performance of the system in terms of output power (*P*_*o*_) and efficiency (*η*) when adding another transmitter coil at ground side.
- Decrease the output power fluctuation in the distance between the two transmitter coils by controlling the operating frequency and insert the magnetizable concrete of the magnetic design.
- Comparing the output power improvement cases in terms of weight, size, and cost, and reaching the most cost-effective case.

The rest of the paper is organized as follows: section “System description and analytical modeling” presents the description and computational model of the proposed design, then the design methodology is presented in section “Design methodology”. An analysis of the transmitter and receiver dimensions, ferrite and shielding dimensions. In section “magnetic design and analysis of dynamic inductive coupler”, the performance analysis of the proposed design in cases of perfect alignment and misalignment circumstances are presented, in addition, the case of having two transmitter and one receiver coil were studied, where an analysis of the mutual inductance between all transmitter and receiver coils *P*_*o*_, and *η* were made at different distances between the two transmitter coils. In section “Power fluctuations improvements” Cases of improving the fluctuating output power at small and large distances between the two transmitter coils were studied, as well as comparing these cases in terms of weight, size and cost. Finally, the results are summarized in section “Conclusion and future work”.

## System description and analytical modeling

**Figure 2:**
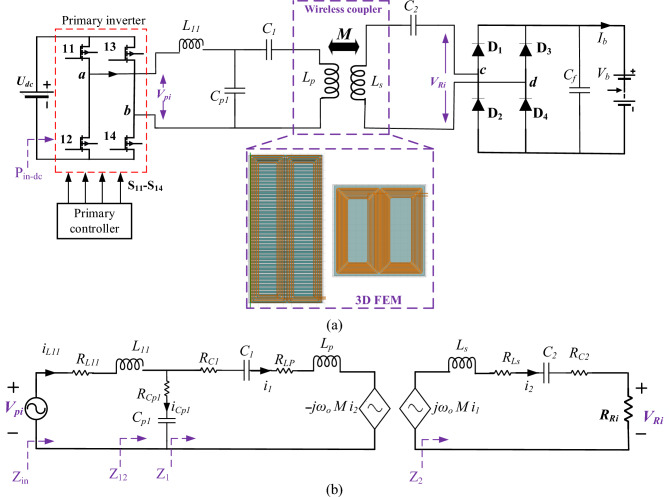
Wireless charging system, (**a**) schematic diagram of DDT/DDR with an LCC-S compensation, and (**b**) equivalent circuit model.

The wireless charging system consists of two sides that are electrically isolated from each other, one of which is the ground side that contains the power transmission station, which includes power transmission coil (*L*_*p*_) connected with a primary LCC compensation network (*L*_*11*_, *C*_*p1*_, *C*_*1*_), that are fed through a full-bridge high-frequency (HF) inverter with a controller. On the other side, the power is received through a power pad that is hung beneath the car. This pad consists of power receiving coil (*L*_*s*_), a secondary series compensation network (*C*_*2*_), and a rectifier to feed the electric vehicle battery, as shown in Fig. 2a. Power is transmitted from the ground to the EV by means of electromagnetic induction, through the mutual inductance (*M*) between the transmitter and receiver coils. The inverter that operates at the resonant frequency (*f*_*o*_) with duty ratio (*D*) ranging from 0 to 1 is fed with a low-frequency Dc voltage. This inverter converts the DC voltage into a high frequency AC voltage (*V*_*pi*_) to feed the primary compensation network. In light of the Fourier series, the main component of the inverter voltage can be calculated as in (1). To obtain the mathematical modeling of the proposed charging system, the equivalent circuit model of the system was used as depicted in Fig. 2b.

The EV battery is represented as resistive load (*R*_*L*_). Taking into account the series compensation capacitor at receiver side as well as the rectifier, the equivalent AC resistance value can be calculated at the output of the compensation network as given in (2)^21^.

1 $$V_{pi} = \frac{2\sqrt 2 }{\pi } U_{dc} {\text{ sin}}\left( {\frac{\pi D}{2}} \right)$$

2 $$R_{Ri} = \frac{8}{{\pi^{2} }}R_{L}$$

The primary LCC compensation network is designed from an LC resonant tank consisting of an inductor (*L*_*11*_) operating at resonance with a capacitor (*C*_*p1*_) and connected in series with a capacitor (*C*_*1*_). The LC resonant tank makes sure that a constant output current is obtained. This constant current passes through the transmitter coil, which is the purpose of the primary LCC compensation. The parallel capacitor (*C*_*p1*_) inside the resonance tank resonates with the capacitor (*C*_*1*_) and the inductance of the transmitter coil (*L*_*p*_) that connected together in series. The series capacitor on the receiver side (*C*_*2*_) resonates with the inductance of the receiver coil (*L*_*s*_). The compensation elements can be estimated according to the resonant frequency (${\omega }_{o}$) which is given in (3)^10^.

3 $$\omega_{o}^{2} = \frac{1}{{L_{s} C_{2} }} = \frac{1}{{L_{11} C_{p1} }} = \frac{1}{{\left( {L_{p} - L_{11} } \right)C_{1} }}$$

where $${\omega }_{o}=2\pi {f}_{o}$$ (rad/s).

The mathematical representation of the LCC-S compensation model was performed and the impedance equations for the circuit components shown in Fig. 1b were derived^11^. The total input impedance of the compensation (*Z*_*in*_) is also deduced as given in (4). The quantities given on the image "*R*_*xx*_" represent the internal equivalent resistances of the compensation parameters or the resistances of the power transmitting and receiving coils, for example (*R*_*C1*_) is the equivalent resistance of the capacitor (*C*_*1*_), and so on.

4 $$Z_{in} = Z_{L11} + \frac{{Z_{1 } Z_{Cp1} }}{{Z_{1} + Z_{Cp1} }} = R_{in} + jX_{in}$$

The current coming out of the inverter (*i*_*L11*_), the current of the receiving side (*i*_*2*_), and the battery charging current (*I*_*b*_) can be calculated as given in (5)^22,23^.

5 $$i_{L11} = \frac{{V_{pi} }}{{Z_{in} }}, i_{2} = \frac{{j \omega_{o} M i_{1} }}{{Z_{2} }}, I_{b} = \frac{2\sqrt 2 }{\pi } i_{2}$$

Knowing the self-inductance of both the transmitting and receiving coils, as well as the mutual inductance between them, the coupling coefficient (*k*) can be calculated as in (6). This coupling factor changes due to the change of many factors, including the variation of the air gap distance, the occurrence of misalignment, etc. The quality factor of the receiving circuit can be calculated as given in (7). By means of the coupling coefficient, the quality factor of the receiving circuit, the current passing through the transmitter coils and its self-inductance, the power transmitted from the ground side to the vehicle side can be calculated at the resonant frequency as given in (8)^11,24^. Transportation efficiency (*η*) can also be calculated as given in (9)^14^.

6 $$k = \frac{M}{{\sqrt {L_{p} L_{s} } }}$$

7 $$Q_{2} = \frac{{\omega_{o} L_{s} }}{{R_{Ri} }}$$

8 $$P_{out} = \omega_{o} i_{1}^{2} k^{2} L_{p} Q_{2}$$

9 $$\eta = \left( {\frac{{R_{in} }}{{R_{L11} + R_{in} }}} \right) \left( {\frac{{R_{r} }}{{R_{Lp} + R_{r} }}} \right)\left( {\frac{{R_{Ri} }}{{R_{Ls} + R_{Ri} }}} \right) \times 100$$

## Design methodology

**Figure 3:**
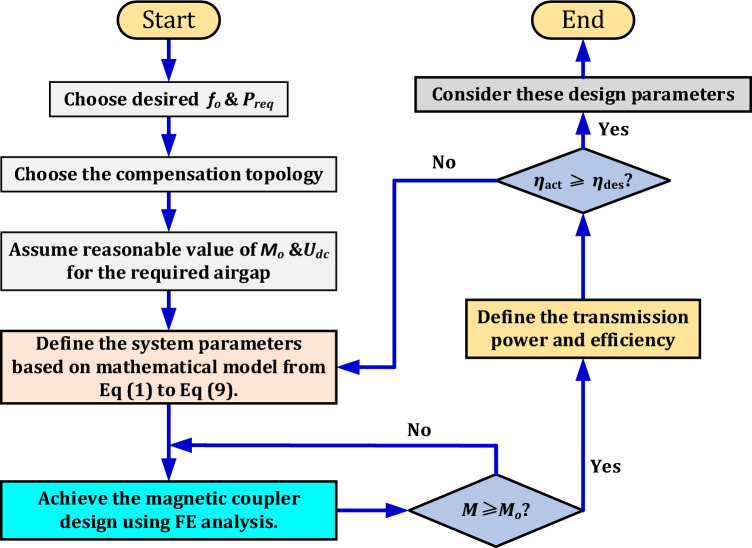
Flowchart to illustrate the proposed design strategy.

The proposed charging system consists of six critical parameters that have to be carefully tuned and designed to achieve the desired power. These parameters are divided into compensation network components (*L*_*11*_, *C*_*p1*_, *C*_*1*_, *C*_*2*_), and magnetic coupler components (*L*_*p*_, *L*_*s*_) as depicted in Fig. 2. A clear methodology for designing these parameters was established by creating a flowchart to illustrate the design steps as shown in Fig. 3.

This flowchart shows that the first stage of designing the proposed charging system is to choose the operating frequency (*f*_*o*_) and the power level to be achieved (*P*_*req*_) which represents the output power of the proposed system (*P*_*o*_), in addition to the airgap distance between the transmitting and receiving coils and the appropriate compensation topology. The operating frequency was determined by the range (79–90 kHz) recommended by the Society of Automotive Engineers (SAE J2954/3) standard version 3, where the nominal frequency of 85 kHz is chosen^25,26^. In addition, the distance of the airgap was determined from the same standard, where the ground clearance suitable for charging electric vehicles was defined as being divided into three classes Z1, Z2, and Z3. The proposed design's air gap of 250 mm was chosen to match the larger airgap (Z3-class), and the flush-ground installation will be taken into account that means, the airgap distance is the same as the ground clearance. The transmitter pad was chosen at the same level as the ground for ease of installation and maintenance. This also provides protection for the transmitter pad from vandalism. The power level to be achieved was determined to be 200 kW and the LCC-S compensation topology was selected in the analysis.

Using LCC compensation network at transmitter achieves a constant current in the transmitting coil at resonance, as well as it works to make the resonant frequency independent of changes in both the coupling coefficient and the load. Therefore, the system can work at a constant switching frequency and zero-voltage switching (ZVS) can be achieved over a wide load range. Additionally, its properties enable several transmitters to be connected to one inverter which facilitates the control process. In addition, it achieves higher transmission efficiency by minimizing the reactive power resulting from the high frequency inverter^10,11,22^. A compensating network with a lower-order (series compensation) was chosen to meet the compact space requirements of the receiver side. Because of the constant voltage behavior at resonance, the series compensation is safe, therefore it is selected at receiver.

**Figure 4:**
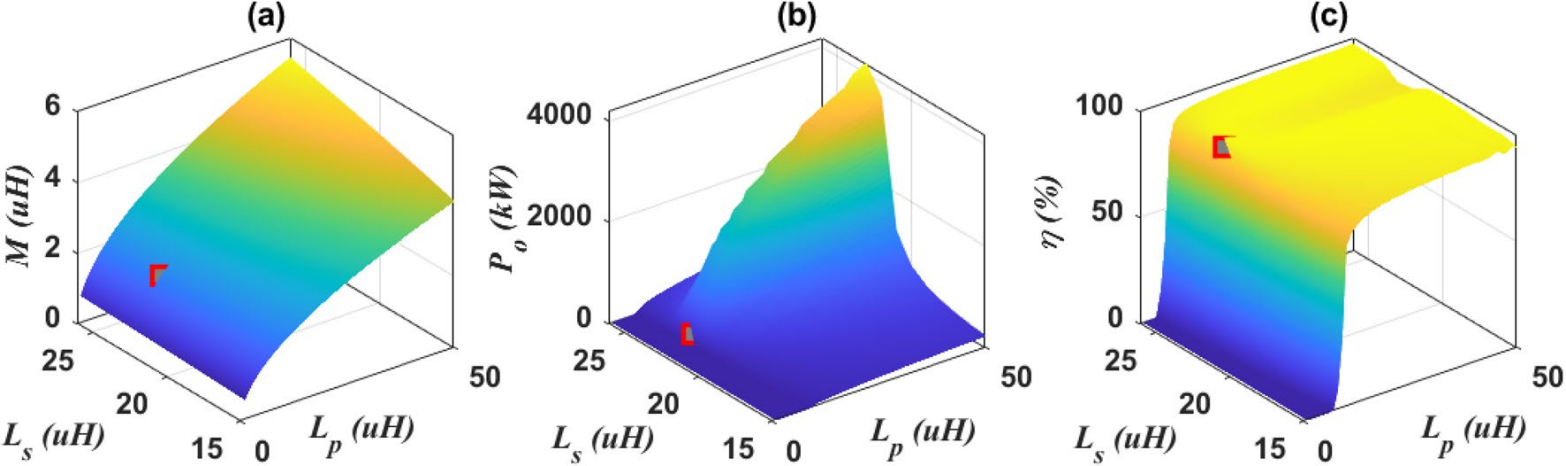
Estimation of magnetic coupler parameters, (**a**) *L*_*p*_*, L*_*s*_ vs. *M*, (**b**) *L*_*p*_*, L*_*s*_ vs. *P*_*o*_, and (**c**) *L*_*p*_*, L*_*s*_ vs. *η*.

Based on what has been determined, a value of mutual inductance (*M*) is imposed based on the chosen air gap distance.The next stage of the design is to find the inductance values of the magnetic coupler by means of equations from (1) to (19). Assuming the initial values of the mutual inductance (*M*) = 1 μH, the battery voltage (*V*_*b*_) = 800 V, and the DC input voltage of the inverter (*U*_*dc*_) = 800 V, the self-inductance of the transmitter (*L*_*p*_) and receiver (*L*_*s*_) coils can be estimated as in Fig. 4a. The output power (*P*_*o*_) and transmission efficiency (*η*) are analyzed according the self-inductances of the two coils as depicted in Fig. 4b and c, respectively. It was concluded that a power value of 200-kW can be transferred with an efficiency of 91.88% at values of self- and mutual-inductances of *L*_*p*_ = 7.4 μH, *L*_*s*_ = 22.47 μH, and *M* = 1.935 μH.

Once the parameters of the magnetic coupler are obtained, the next stage for the design is to perform an analysis of both the coils and the magnetic material by means of finite element analysis (FEA) to obtain the self and mutual-inductance that achieves the required power level. More details about the system analysis are introduced below.

## Magnetic design and analysis of dynamic inductive coupler

When constructing inductive charging couplers used in stationary charging, design specifications are taken into account that help improve the transfer of optimal power with high efficiency during the distance of an air gap and over a small scale of misalignments. In case of in-motion charging of electric vehicles, the value of the net energy transferred from the transmitter coil to the receiving coil must be taken into account while the vehicle carrying the receiving coil passes along the transmission path. Also, the transmitter coil must have a dedicated lane in the road, and it must be designed to withstand environmental conditions more severe than those facing the stationary charging system.

### Rectangular, circular and double-D comparison

If a comparison is made between square and circular coils, it becomes clear that the former gives better performance in terms of the coupling coefficient and also gives a higher tolerance in lateral misalignment case^27^. When comparing rectangular coils with DD coils, it is noted that the coupling properties of the latter are higher than those of the former^26^. Shielding of the EV chassis is one of the most important considerations due to the natural misalignment in the driving direction. Preliminary work of coupler designs showed that circular coils had better performance than DD coils. However, EV shielding requirements have a significant impact on the performance characteristics of circular coils. When adding the shielding of the EV chassis, the power generated by the DD coils is almost unaffected, while the power of the circular coils is reduced by 50%. The merits of circular coils are significantly reduced when modifications are made to the magnetic design to accommodate shielding. Even after the shielding problem was resolved, the sensitivity of the circular coils remained a concern regarding the many uncertainties associated with the practical system. So, the robustness of the DD configuration has priority over the low mass of the circular configuration.

Additionally, there are practical reasons to prefer the DD configuration for high-power in-motion systems. The magnetic coupler can be configured with multiple litz wires connected in parallel, thus decreasing the voltage drop of it and provide flexibility. This flexibility of parallel litz wire makes DD coil manufacturing easier. In the circular configuration, the outer wire would achieve inductance larger than the inner wire. Therefore, the performance of the system will decrease as a result of an unbalance in the current caused by an unbalance in the inductance. Conversely, in the DD configuration, multiple litz wires are moved during the winding process, resulting in improved inductance and current balance between the wires^19^. Therefore, the DD coil configurations is used in this study because of its advantages.

### Design of 200-kW DD-DD dynamic coupler

The DD inductive pad was constructed on both transmitter and receiver sides due to its merits of high efficiency, large tolerance for misalignment, and simplicity of design. A 3D finite-element models (3D FEMs) for the proposed magnetic coupler (DD-transmitter pad (DDTP), and DD-receiver pad (DDRP)) are developed, designed, and analyzed. Magneto-static solution of Maxwell software is utilized to evaluate the parameters of magnetic coupler. In addition to the use of commercial ferrite N87 for both transmitter and receiver pad, which is the common and ideal choice for inductive power transfer (IPT) applications due to its features including low electrical conductivity and high magnetic permeability, which leads to reducing losses at high operating frequencies^28^. After obtaining the parameters of the magnetic coupler, they can be considered as constants, and thus FEM is used to obtain the coil, ferrite, and shielding dimensions, number of turns, and space between turns (*pitch*) for each of the transmitting and receiving coils. Because of space limitations, the transmitter width (*W*_*cT*_) is set at 850.9 mm and the receiver width (*W*_*cR*_) is 660 mm and each pad is specified as follows.

**Figure 5:**
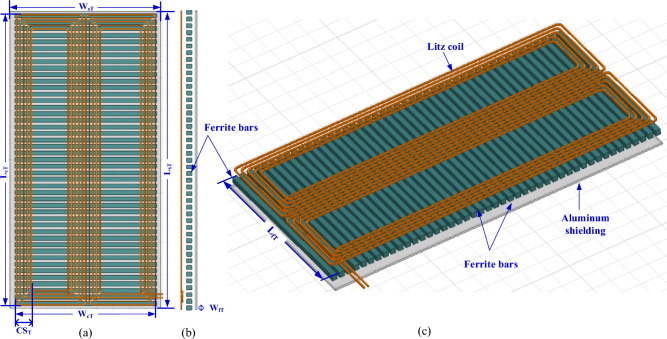
DD coil-based transmitter pad (DDTP) for 200-kW DIPT system: (**a**) top view, (**b**) side view, and (**c**) 3D-model.

A 3-D model of the DD transmitter pad (DDTP) pad was developed, which consists of a transmitter coil made of litz wire and formed as single turn coil containing five parallel wires, each with a diameter of 6.54 mm, to reduce the resistance of the coils, provide balance for the current between the wires, and reduce the total inductance as depicted in Fig. 5. Under this coil with a separation of 10 mm, a string of 45 bars of ferrite N87 is placed in parallel. Each string contains standard ferrite cores (I 200 × 25 × 10) connected in series. So, the dimensions of each string are 860.9 × 25 × 10 mm^3^. These bars are separated by a horizontal distance (*D*_*f*_) and used to confine the magnetic field between the transmitter and receiver coils, therefore they reduce the leakage field.

**Figure 6:**
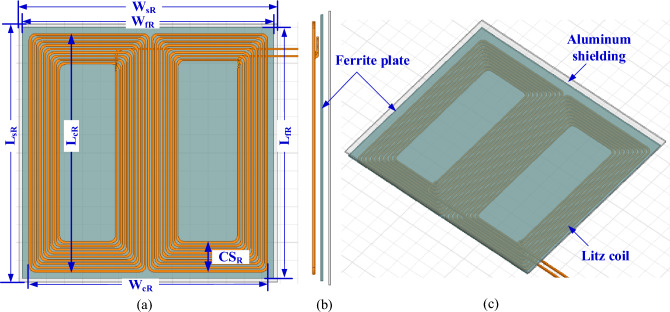
DD coil-based receiver pad (DDRP) for 200-kW DIPT system: (**a**) top view, (**b**) side view, and (**c**) 3D-model.

On the other side of the charging coupler, a DD receiver pad (DDRP) pad was used. It also consists of a receiver coil that contains three turns, each of which consists of three parallel wires, so that the total wires in the receiving coil are 9 parallel wires, each of which is 5 mm in diameter, to reduce the resistance of the coils, provide balance for the current between the wires, and reduce the total inductance as shown in Fig. 6. A ferrite plate was placed with a thickness of 5 mm above the receiver coil with a distance of 8 mm to keep from leaking large amounts of magnetic field.

**Figure 7:**
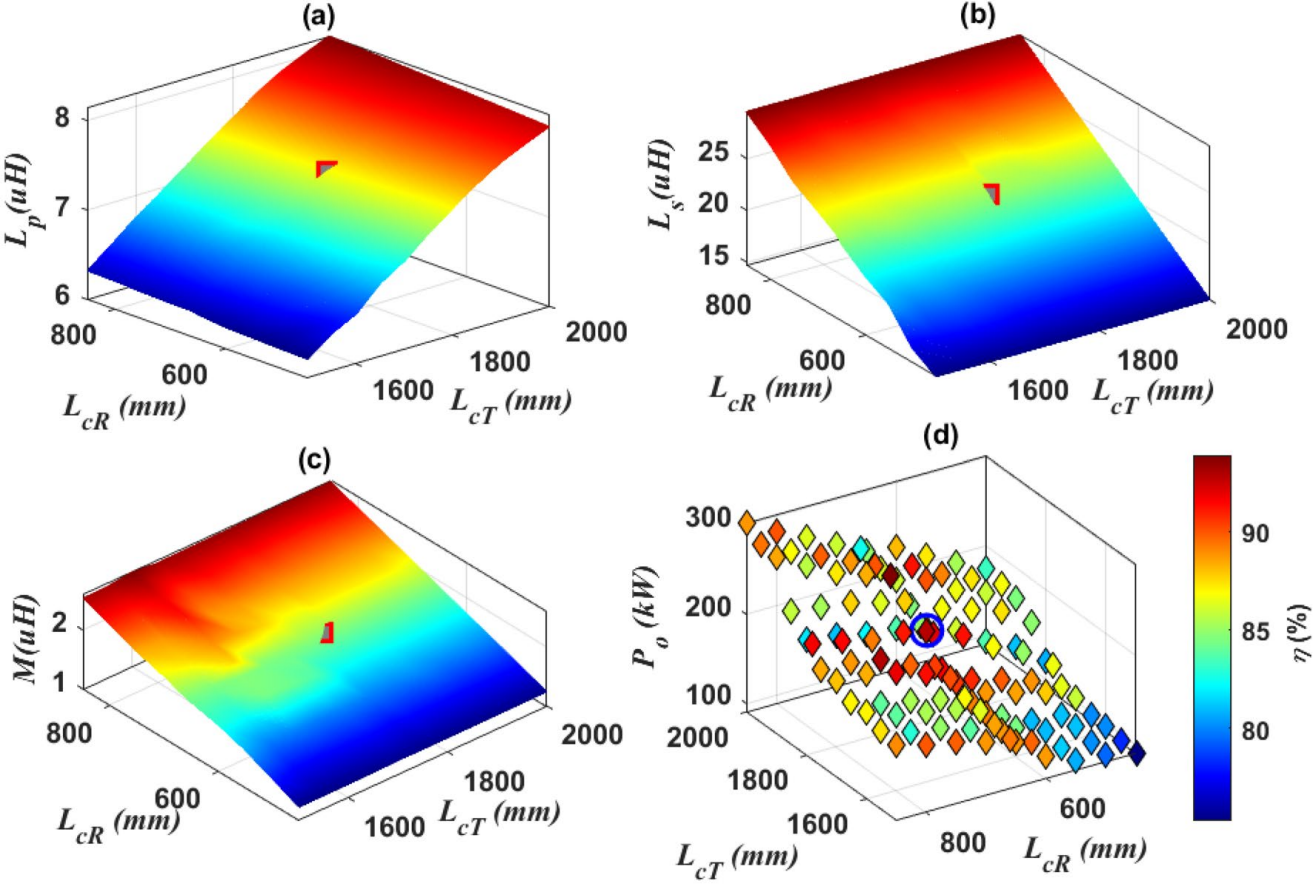
Analysis of DDTP-DDRP dimensions, (**a**) relationship between *L*_*cT*_, *L*_*cR*_, and *L*_*p*_, (**b**) relationship between *L*_*cT*_, *L*_*cR*_, and *L*_*s*_, (**c**) relationship between *L*_*cT*_, *L*_*cR*_, and *M*, and (**d**) relationship between *L*_*cT*_, *L*_*cR*_, *P*_*o*_ and *η*.

Therefore, it becomes clear that the variables taken into account are the length of transmitter (*L*_*CT*_), and length of receiver (*L*_*cR*_), as well as the distance between each turn of the transmitter (*pitch-*_*T*_) and receiver (*pitch-*_*R*_), in addition to the distance between the ferrite bars (*D*_*f*_) on the transmitter side. To obtain the magnetic parameters (*L*_*p*_*, L*_*s*_*, M*) that achieve an output power of 200-kW with highest efficiency, the length of the transmitting coil (*L*_*cT*_) was changed from 1500 to 2000 mm by step of 50 mm, and at each step the length of the receiving coil (*L*_*cR*_) was changed from 450 to 850 mm by step of 50 mm. A relationship was drawn between the length of the transmitting coil *L*_*cT*_ and the length of the receiving coil* L*_*cR*_, with coupler parameters as depicted in Fig. 7.

The self-inductance of the transmitter *L*_*p*_ and receiver coils* L*_*s*_, and mutual inductance* M* vs.* L*_*cT*_ and *L*_*cR*_ are represented in Fig. 7a–c respectively. It is clear that, expanding the lengths of the coils leads to an increase in both self and mutual inductance for both coils. The output power* P*_*o*_ and transmission efficiency (*η*) were investigated when the length of the transmitter and receiver coils changed, and the relationship between them was drawn as depicted in Fig. 7d. As the coil lengths increase, the *P*_*o*_ and *η* increase. The operating point is marked in red and blue, which gives the appropriate length for each of the transmitter and receiver coils that gives the required power and efficiency and listed in Table 1.

**Table 1:** Magnetic parameters, electrical specifications, and dimensions of DDTP and DDRP.

Parameter	Value	Parameter	Value
*L*_*p*_	7.4 μH	*CS*_*T*_	93.5 mm
*L*_*s*_	22.47 μH	*CS*_*R*_	80 mm
*M*	1.935 μH	*Pitch-*_*T*_	15.2 mm
*L*_*11*_	1.63 μH	*Pitch-*_*R*_	4.37 mm
*C*_*p1*_	2.14 μF	*L*_*fT*_	860.9 mm
*C*_*1*_	0.607 μF	*L*_*fR*_	670 mm
*C*_*2*_	0.156 μF	*W*_*fT*_	25 mm
*V*_*b*_	800 V	*W*_*fR*_	670 mm
*U*_*dc*_	800 V	*D*_*f*_	15 mm
*L*_*cT*_	1778 mm	*L*_*sT*_	1788 mm
*L*_*cR*_	660 mm	*L*_*sR*_	700 mm
*W*_*cT*_	850.9 mm	*W*_*sT*_	898.5 mm
*W*_*cR*_	660 mm	*W*_*sR*_	700 mm

**Figure 8:**
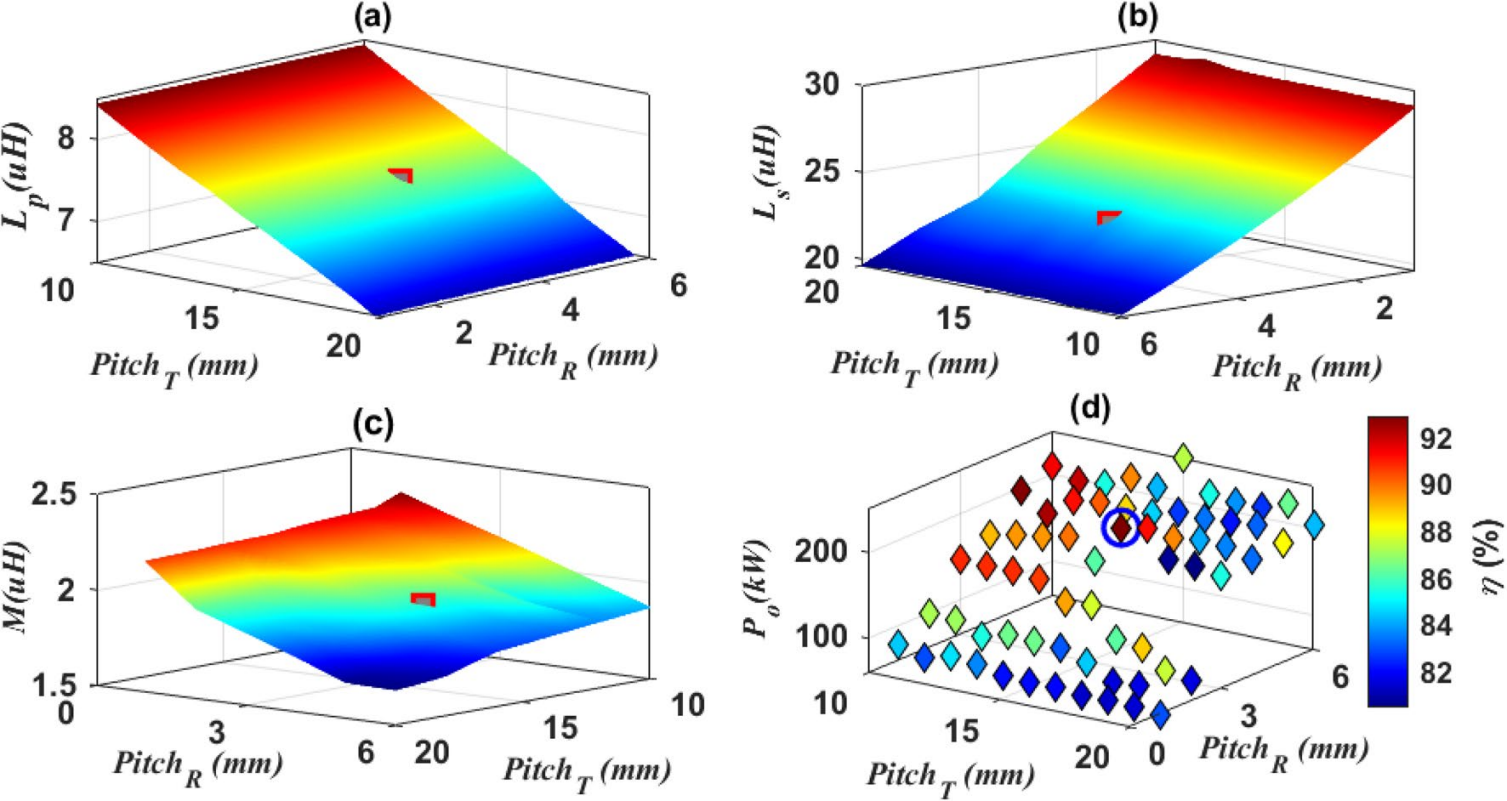
Analysis of DDTP-DDRP dimensions, (**a**) relationship between *pitch-*_*T*_, *pitch-*_*R*_, and *L*_*p*_, (**b**) relationship between *pitch-*_*T*_, *pitch-*_*R*_, and *L*_*s*_, (**c**) relationship between *pitch-*_*T*_, *pitch-*_*R*_, and *M*, and (**d**) relationship between *pitch-*_*T*_, *pitch-*_*R*_,* P*_*o*_ and *η*.

After obtaining the transmitter and receiver lengths, their values mentioned in Table 1 were fixed in the rest of the analysis, then the effect of changing *pitch-*_*T*_ and *pitch-*_*R*_ was studied. The *pitch-*_*T*_ was changed from 10 to 20 mm with a step of 1 mm and at each step the *pitch-*_*R*_ was changed from 1 to 6 mm. At each step, the values of *L*_*p*_, *L*_*s*_, *M, P*_*o*_ and *η* were calculated as shown in Fig. 8. It was concluded that with the increase of *pitch-*_*T*_ and *pitch-*_*R*_, the value of the self- inductance of each of the two coils decreases, while an improvement occurs in the mutual inductance and the output power. The *pitch-*_*T*_ and *pitch-*_*R*_ that give reasonable *M*, *η* and desired *P*_*o*_ are estimated as marked in Fig. 8d and are presented in Table 1.

**Figure 9:**
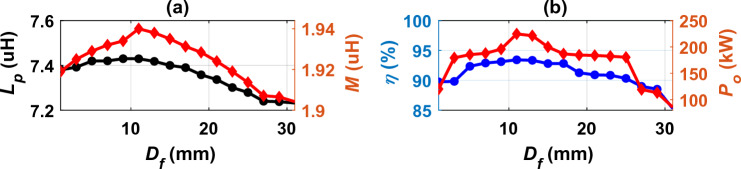
Analysis of DDTP-DDRP dimensions, (**a**) *D*_*f*_ vs. *L*_*p*_ and *M*, (**b**) *D*_*f*_ vs. *P*_*o*_ and *η*.

Distance between ferrite *D*_*f*_ is another variable that has been studied. The *D*_*f*_ was changed from 1 to 31 mm in steps of 2 mm. The values of *L*_*p*_, *M*, *P*_*o*_ and *η* were calculated and plotted as shown in Fig. 9. With a slight increase in *D*_*f*_, all variables increase by a very small amount, but with a significant increase in *D*_*f*_, these variables decrease dramatically. In addition, with the increase in *D*_*f*_, the ferrite area decreases, and accordingly, the ferrite weight used in the design decreases. As a result, the cost and weight of the system is reduced while achieving the required power with the highest efficiency. The *D*_*f*_ value was chosen at 15 mm, which gives the best *M* that achieves 200-kW power output with an efficiency of 91.88%. All variables of DDTP-DDRP system are presented in Table 1 including magnetic and electrical parameters as well as coils dimensions.

### Performance analysis of DDTP-DDRP

**Figure 10:**
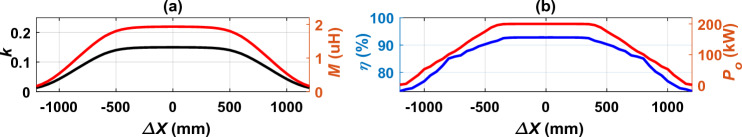
Performance of DDTP-DDRP system in driving direction, (**a**) *k* and* M* vs. *∆X*, and (**b**) *η* and* P*_*o*_ vs. *∆X*.

After achieving the appropriate dimensions of the DDTP-DDRP design, the performance of the system was investigated in both cases of perfect alignment and linear misalignment by using MATLAB Simulink. The X-axis is considered as the driving direction and represented by (*∆X*), while the Y-axis is the direction of the linear misalignment occurrence (lane direction) and represented by (*∆Y*). The performance of the system was studied at fully alignment with the movement in the driving direction (*∆X*). The value of the mutual inductance *M* and the coupling coefficient *k* were represented with the movement of the vehicle from − 1200 to 1200 mm on the driving axis *∆X* as depicted in Fig. 10a. The output power (*P*_*o*_) value corresponding to each value of *M* was calculated and the transmission efficiency (*η*) was calculated and both represented as shown in Fig. 10b. When the electric vehicle is moving in driving direction (*∆X*), the value of mutual inductance (*M*) remains constant as long as the vehicle coil is perfectly aligned with the transmitter coil. The value of *M* decreases when the vehicle enters or exits the transmitter pad. This is due to the occurrence of a lack of alignment in the X-axis direction where the value of *M* begins to decrease at *∆X* =  ± 300 mm and continues to decrease gradually until the value of *M* reaches zero in the event of complete misalignment in *∆X*.

Similarly, the output power relationship is with the movement of the vehicle, where the *P*_*o*_ value is 200-kW at *∆X* =  ± 350 mm, after which it gradually decreases until it reaches its lowest value at the position ± 1200 mm. This system achieves a transmission efficiency of 91.88%, as a maximum efficiency when fully lined up, and with the occurrence of movement in the driving direction (*∆X*), this efficiency decreases to reach 73.14%.

**Figure 11:**
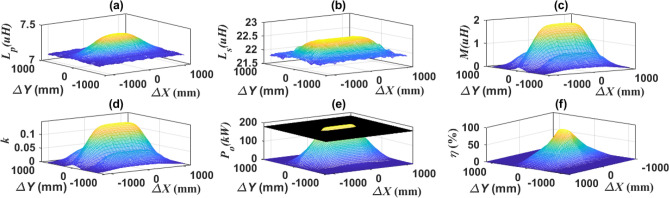
Performance of DDTP-DDRP system under lateral misalignments, (**a**) *L*_*p*_ vs. *∆X* and *∆Y*, (**b**) *L*_*s*_ vs. *∆X* and *∆Y*, (**c**) *M* vs. *∆X* and *∆Y*, (**d**) *k* vs. *∆X* and *∆Y*, (**e**) *P*_*o*_ vs. *∆X* and *∆Y*, and (**f**) *η* vs. *∆X* and *∆Y*.

At misalignment in lane direction, the self-inductance of the transmitter (*L*_*p*_), self-inductance of the receiver (*L*_*s*_), mutual inductance (*M*) and coupling factor (*k*) were represented in case of the vehicle moving in *∆X* from − 1200 to 1200 mm, and also when there was linear misalignment in the Y-axis direction from − 800 to 800 mm. The relationship of *L*_*p*_, *L*_*s*_, *M*, *k*, *P*_*o*_, and *η* in the driving direction (*∆X*) and lane direction (*∆Y*) are represented in Fig. 11. From Fig. 11a, it is clear that the value of *L*_*p*_ ranges from 7.11 to 7.38 μH and the percentage change is 3.65%. So, it is almost constant and does not change with the movement of the vehicle, whether this movement is in the driving direction or in lane direction (*∆Y*). Figure 11b shows that the value of *L*_*s*_ is decreased with the occurrence of misalignment in the Y-axis direction. This reduction is within small range (3.24%) that it can be overlooked and not taken into account.

Figure 11c represents the relationship of mutual inductance (*M*) with the motion in the driving direction and the lateral misalignment direction. The highest value of *M* is at perfect alignment (*∆Y* = 0). This value remains constant as long as the center of the receiving coil is exactly aligned with the center of the transmitter coil. In case of increasing misalignment, the high *M* value begins to decrease gradually until it reaches small values at distance of ± 200 mm to ± 350 mm, due to the movement of receiving coil away from the middle of the transmitter coil. The value of *M* remains decreasing until the receiving coil approaches the external coil side of the transmitter coil, and then the value of *M* begins to increase gradually until it reaches somewhat high values at the distance of ± 350 mm to ± 550 mm. As the receiving coil moves farther from the transmitter, the value of *M* decreases until it reaches zero. Since *L*_*p*_ and *L*_*s*_ were taken as constant values that are not affected by the movement of the vehicle, the value of the coupling factor (*k*) follows the value of the mutual induction (*M*) between the two coils as shown in Fig. 11d.

The output power profile (*P*_*o*_) is represented in Fig. 11e. In the scenario of movement in driving direction (*∆X*), when the EV enters the charging path, the *P*_*o*_ begins to increase gradually until it reaches its highest value when fully lined up with the transmitter coil at a distance from − 350 to + 350 mm. In addition, whenever the EV moves out of the charging path, the *P*_*o*_ gradually decreases until it reaches its lowest value. The *P*_*o*_ value starts to decrease at a distance of ± 400 mm. When moving in lane direction (*∆Y*), the maximum value of *P*_*o*_ is obtained at perfect alignment (*∆Y* = 0), and this *P*_*o*_ is greatly reduced when a linear misalignment occurs in lane direction (*∆Y* =  ± 100 mm). The transmission efficiency *η* is introduced in Fig. 11f, where the highest efficiency occurs at perfect alignment and then gradually decreases until it reaches its lowest value in ∆*Y* direction. In *∆X* direction, the percent change in the value of efficiency from perfect alignment to maximum misalignment is 21%. The misalignment points on the X-axis and the Y-axis were deduced, as well as the transmission efficiency *η* at three power levels: 100% of *P*_*o*_, 90%of *P*_*o*_, and 80% of *P*_*o*_. The level 90% of *P*_*o*_ is represented in Fig. 11e. These values are summarized in Table 2.

**Table 2:** Movement states of driving and lane directions.

Moving direction	Driving direction (*∆X*)		Lane direction (*∆Y*)	
	*∆X*	*η* (%)	*∆Y*	*η* (%)
100% of *P*_*o*_	*0*	91.8	0	91.8
90% of *P*_*o*_	± 472.7 mm	90.8	± 7.07 mm	89.9
80% of *P*_*o*_	± 569.7 mm	87.45	± 13.13 mm	88.14

### Double transmitters-single receiver system

Unlike stationary charge, in-motion charging system can consist of multiple transmitters with a single receiver (MTSR) or multiple receivers (MTMR). Using MTMR system is suitable for light- and heavy-duty EVs, achieve high power transmission with appropriate efficiency, and reduce the excess voltage on the system semiconductors. Using multiple receivers improves the total mutual inductance between the transmitter and the receiver, which helps the system to transmit higher power. In addition, providing a modular design for the receiver pad, which achieves a high scalability for the charging system.

**Figure 12:**
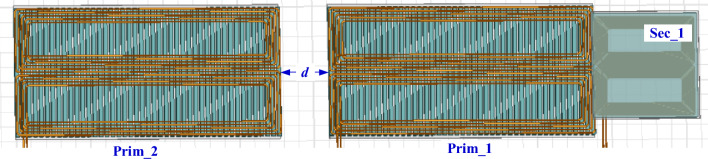
Top view of DTSR coupler for 200-kW DIPT system.

Two identical transmitter pads are designed at ground side, separated by a horizontal distance “*d*”, as depicted in Fig. 12. While single receiver pad was suspended in the EV. This design is used to study the appropriate distance between the two transmitter coils “*d*” that gives the best possible power transmission with highest efficiency and minimize the power pulsation.

**Figure 13:**
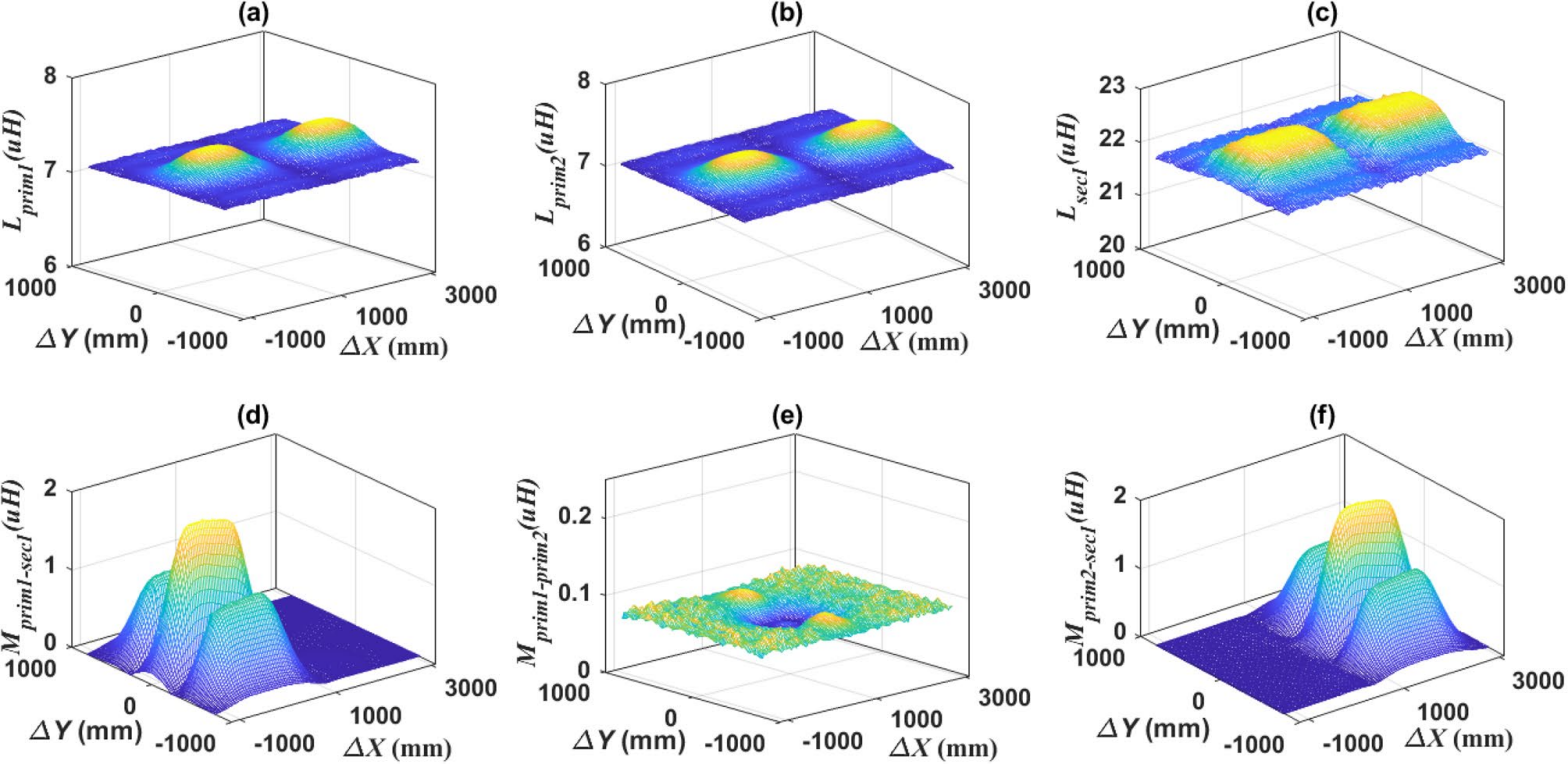
Performance of DTSR system at *d* = 50 mm and under lateral misalignments, (**a**) *L*_*prim1*_ vs. *∆X* and *∆Y*, (**b**) *L*_*prim2*_ vs. *∆X* and *∆Y*, (**c**) *L*_*sec1*_ vs. *∆X* and *∆Y*, (**d**) *M*_*prim1–sec1*_ vs. *∆X* and *∆Y*, (**e**) *M*_*prim1–prim2*_ vs. *∆X* and *∆Y*, and (**f**) *M*_*prim2-sec1*_ vs. *∆X* and *∆Y*.

The performance of the double transmitter-single receiver (DTSR) system has been studied in different misalignment cases, whether in the driving direction (*∆X*-direction) or the lane direction (*∆Y*-direction). The magnetic parameters (*L*_*prim1*_, *L*_*prim2*_, *L*_*sec1*_, *M*_*prim1–sec1*_, *M*_*prim1–prim2*_, and *M*_*prim2–sec1*_) of the DTSR system at small horizontal distance (*d* = 50 mm) between the two transmitters were extracted, when the vehicle is moving from − 1200 to 3100 mm in the driving direction (*∆X*), while the misalignment is from − 800 to 800 mm in the lane direction (*∆Y*) as indicated in Fig. 13.

From Fig. 13a and b, it can be seen that the self-inductance value of the two transmitting coils (*L*_*prim1*_, *L*_*prim2*_) ranges from 7.107 to 7.409 μH and the percentage change is 4.05%. While the self-inductance of the receiving coil (*L*_*sec1*_) ranges from 21.83 to 22.61 μH and the percentage change is 3.44%. It was also noted that the mutual inductance between the two transmitter coils (*M*_*prim1–prim2*_) ranged from 0.0951 to 0.10 μH and the percentage change is 5%. Therefore, it can be concluded that the change in the self-inductance of the transmitter and receiver coils (*L*_*prim1*_, *L*_*prim2*_, and *L*_*sec1*_) is very small and can be ignored. Therefore, *L*_*prim1*_, *L*_*prim2*_, and *L*_*sec1*_ are considered constant at 7.4 μH, 7.4 μH, and 22.47 μH respectively. The variation in the mutual inductance between the two transmitter coils (*M*_*prim1–prim2*_) can be neglected. Moreover, the value of *M*_*prim1–prim2*_ is very small compared with the base value of inductance (1.973 μH), therefore, it can be neglected. Mutual inductance between prim1–sec1 (*M*_*prim1–sec1*_) and prim2–sec1(*M*_*prim2–sec1*_) can be taken into account.

**Figure 14:**
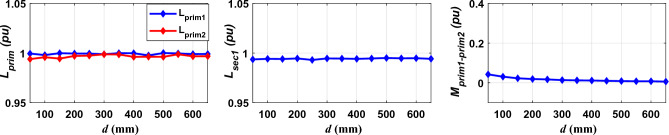
The relationship between *L*_*prim1*_, *L*_*prim2*_, *L*_*sec1*_, and *M*_*prim1–prim2*_ and the horizontal distance between the two transmission coils “*d*”.

To make sure of this, the per unit (PU) system was used to normalize all these values. Where all the mutual inductance were assigned to the base value of 1.973 μH, the self-inductance of the two transmitter coils (*L*_*prim1*_, *L*_*prim2*_) was assigned to the base value of 7.409 μH, and the self-inductance of the receiving coil (*L*_*sec1*_) was assigned to the base value of 22.61 μH. The values of (*L*_*prim1*_, *L*_*prim2*_, *L*_*sec1*_, and *M*_*prim1–prim2*_) were also measured at variable horizontal distances between the two transmitting coils, changing from *d* = 50 to *d* = 650 mm, as depicted in Fig. 14. From this normalization, it can be concluded that the percentage change in the self-inductance of the two transmitter coils with distance “*d*” is equal to (0.3%) and the percentage change in the self-inductance of the receiver coil with distance “*d*” is equal to (0.2%), and the percentage change in the mutual inductance between the two transmitter coils with distance “*d*” is equal to (4%). In addition, the mutual inductance between the two transmitter coils decreases significantly as the horizontal distance “*d*” between them increases.

Therefore, the variation of *L*_*prim1*_, *L*_*prim2*_, *L*_*sec1*_, and *M*_*prim1–prim2*_ between the maximum and minimum values, whether with the distance “*d*” or due to the occurrence of misalignment in the lane direction (*∆Y*), is considered a very small change that can be neglected. The value of *M*_*prim1–prim2*_ is very small therefore, it can be ignored. While the values of *L*_*prim1*_, *L*_*prim2*_, and *L*_*sec1*_ are considered constant in the remaining analysis and do not change with the movement of the vehicle.

Similarly, when the horizontal distance “*d*” between the two transmitter coils is increased to 330 mm and 660 mm, then the different magnetic parameters of the DTSR system (*L*_*prim1*_, *L*_*prim2*_, *L*_*sec1*_, and* M*_*prim1–prim2*_) were extracted. It can be concluded that; the change in the value of self-inductance for both transmitter and receiver coil is very small, so these values can be considered constant for both coils and do not change with the movement of the vehicle. While the value of the change in the mutual inductance between the two transmitter coils are very small, so it can be neglected. Mutual inductance between prim1–sec1 (*M*_*prim1–sec1*_) and prim2–sec1 (*M*_*prim2–sec1*_) can be taken into account.

**Figure 15:**
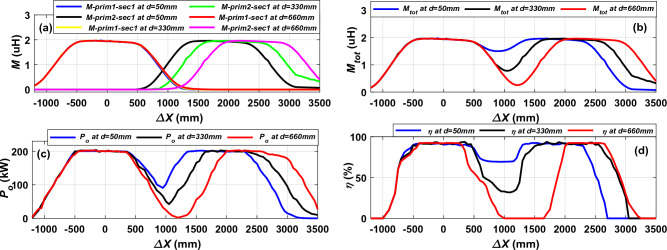
Performance of DTSR system at perfect alignment with different horizontal distance “*d*”, (**a**) *M* vs. *∆X*, (**b**) *M*_*tot*_ vs. *∆X*, (**c**) *P*_*o*_ vs. *∆X*, and (**d**) *η* vs. *∆X*.

The relationships between *M*_*prim1–sec1*_ and *M*_*prim2–sec1*_ are drawn at perfect alignment (*∆Y* = *0*) when the receiver moving in driving direction (*∆X*) as depicted in Fig. 15a. It was found that when the receiver is in alignment with the first transmitter (Prim_1), the value of mutual inductance* M*_*prim1–sec1*_ is as large as possible and remains constant at this value (1.935 μH) until the receiver begins to exit from the Prim_1. Then the *M*_*prim1–sec1*_ gradually decreases until the receiver enters the second transmitter (Prim_2), so the value of mutual inductance *M*_*prim2–sec1*_ begins to increase again until it reaches the highest value (1.935 μH). When the distance *d* is small, the lowest reduction in resultant mutual inductance occurs at *d* = 50 mm and *M*_*tot*_ = 1.5 μH, while as the distance increases, the mutual inductance reduction increases until reaches its highest value at the largest distance *d* = 660 mm and *M*_*tot*_ = 0.25 μH as depicted in Fig. 15b. When the distance *d* = 330 mm the value of resultant mutual inductance will be *M*_*tot*_ = 0.77 μH.

The output power (*P*_*o*_) and efficiency (*η*) values of the DTSR system were plotted at different *d* (50 mm, 330 mm, and 660 mm) as shown in Fig. 15c and d, respectively. The *P*_*o*_ and *η* of the system follow the resultant mutual inductance behavior. When the receiver (Sec_1) moves completely over the Prim_1 in the driving direction from *∆X* = − 500 to 400 mm, the value of the output power* P*_*o*_ and efficiency *η* remain constant at nominal value (200-kW, and 91.88% respectively) in the three cases of horizontal distance *d*. In addition, the power profile at *d* = 50 mm is smoother. When the Sec_1 is fully aligned with the Prim_2, the *P*_*o*_ and *η* remain a constant value, from *∆X* = 1350 to 2300 mm for a horizontal distance *d* of 50 mm, and at *∆X* from 1650 to 2350 for a distance *d* of 330 mm, and at *∆X* from 2000 to 2630 mm for a *d* of 660 mm.

When the receiver moves above the distance between the two transmitter coils, a large fluctuation occurs in the *P*_*o*_ and *η*, where the value of the *P*_*o*_ and *η* decreases to a very large extent at the middle of this distance. At a horizontal distance *d* of 50 mm, the least fluctuation occurs in the output power* P*_*o*_, which is 54.5% at a distance of *∆X* = 950 mm. While the largest fluctuation in the output power* P*_*o*_ occurs at the horizontal distance *d* of 660 mm by 99.5% at *∆X* = 1200 mm. At a distance *d* of 330 mm, the *P*_*o*_ decreases by 78.5% at *∆X* = 1050 mm. The reduction in the transmission efficiency *η* at a distance *d* of 50 mm, 330 mm, and 660 mm is 24.5%, 31.85%, and 100%, respectively. It is concluded that the greater the horizontal distance *d* between the two transmission coils, the greater the fluctuations in the *P*_*o*_ and *η*. The mutual inductance fluctuation is minimum at a small distance *d*, which improves the power and efficiency profiles.

## Power fluctuations improvements

In this section, several techniques are presented to improve the fluctuations in the output power during the passage of the receiving coil over the horizontal distance *d*. When the receiving coil is in the middle of the distance *d*, the lowest value of the output power *P*_*o*_ is transmitted with the lowest efficiency *η*. To improve this *P*_*o*_ and *η*, three techniques are used: the first is operating frequency control, the second is the introduction of magnetizable concrete (MC) for magnetic design, and the third is frequency control with the presence of magnetizable concrete.

### Improvement by operating frequency control

**Figure 16:**
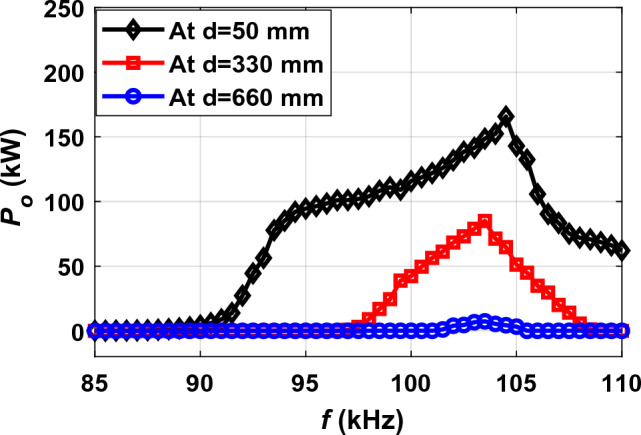
*P*_*o*_ vs. *f* at the middle of distance (*d*) for three different horizontal distances.

Frequency control technique has been applied in the area from the drive direction *∆X* that the power and efficiency begin to fluctuate at *d* of 50 mm (*∆X* from 400 to 1300 mm), at *d* of 330 mm (*∆X* from 400 to 1600 mm), and at *d* of 660 mm (*∆X* from 400 to 1950 mm). The variable frequency (*VF*) value was changed from 85 to 110 kHz with a step of 0.5 kHz. At each frequency value the corresponding *P*_*o*_ value was calculated as shown in Fig. 16. This analysis was made for the three horizontal distances of 50 mm, 330 mm and 660 mm. With the frequency increasing from the fixed frequency (*FF*) of 85 kHz, the value of the output power increases until it reaches its highest value at the variable frequency (*VF*) of 104.5 kHz for a distance *d* of 50 mm, (103.5 kHz) for a distance *d* of 330 mm, and (103.5 kHz) for a distance *d* of 660 mm.

**Figure 17:**
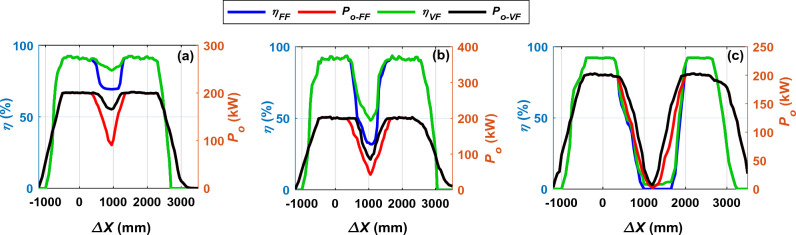
*η*, *P*_*o*_ vs. *∆X* with different operating frequency “*f*”, (**a**) @ *d* = 50 mm, (**b**) @ *d* = 330 mm, and (**c**) @ *d* = 66 mm.

The system is operated at the frequency which gives the maximum *P*_*o*_ for each *d*. In addition, the value of the *P*_*o*_ and *η* are calculated in the region where the fluctuation begins and compared with the results of fixed frequency 85 kHz (*FF*) as shown in Fig. 17. At a horizontal distance *d* of 50 mm, a significant improvement in both output power *P*_*o*_ and efficiency *η* appears as illustrated in Fig. 17a. The output power *P*_*o*_ reaches 165.7 kW at an operating frequency of 104.5 kHz, and despite the frequency increase by a small percentage of 18.66%, the *P*_*o*_ increased by a large percentage of up to 45.15%. In addition, the transmission efficiency *η* has been improved to 82.40% at the same operating frequency by a percentage of 15.95%.

At a horizontal distance *d* of 330 mm, the value of the *P*_*o*_ increases from 42.55 to 84.96 kW when the frequency increases from 85 to 103.5 kHz, as shown in Fig. 17b. The *P*_*o*_ has been improved by 49.94%, but it is still a small value that is not commensurate with the charging process. In addition, the efficiency was improved to reach 48.47%. At a horizontal distance *d* of 660 mm, the output power is improved from 1.91 to 7.29 kW by a percentage of 73.97% when the frequency is raised from 85 to 103.5 kHz, as shown in Fig. 17c. Despite this improvement, the value of the *P*_*o*_ is still too small to suit the in-motion charging of EVs. Moreover, the efficiency was improved by a very small percentage of 2.12%, and this efficiency is very poor. The output power *P*_*o*_ and efficiency *η* values at the middle of distance *d* generated by the improvement process by controlling the operating frequency are summarized in Table 3.

**Table 3:** *P*_*o*_ and *η* values at the middle of distance *d* of the operating frequency control technique.

*d* (mm)	*∆X* (mm)	*M*_*tot*_ (μH)	*FF* (kHz)	*VF* (kHz)	*P*_*o-FF*_ (kW)	*P*_*o-VF*_ (kW)	*%P*_*o- ripple*_	*η*_*FF*_ (%)	*η*_*VF*_ (%)	*%η*_*ripple*_
50	950	1.507	85	104.5	90.87	165.7	45.15	69.25	82.40	15.95
330	1050	0.778	85	103.5	42.55	84.96	49.91	31.94	48.47	34.10
660	1200	0.253	85	103.5	1.91	7.29	73.79	0	2.12	2.12

From the aforementioned, it can be concluded that improving the fluctuation in the output power by controlling the operating frequency is very effective if the horizontal distance *d* is small (50 mm). On the contrary, if the distance *d* is increased to reach the entire length of the receiver (660 mm) or even half of its length (330 mm), controlling the operating frequency will be useless and ineffective. Therefore, it is resorted to inserting the magnetizable concrete (MC) into the magnetic design at the horizontal distance between the two transmitter coils.

### Improvement by magnetizable concrete (MC)

**Figure 18:**
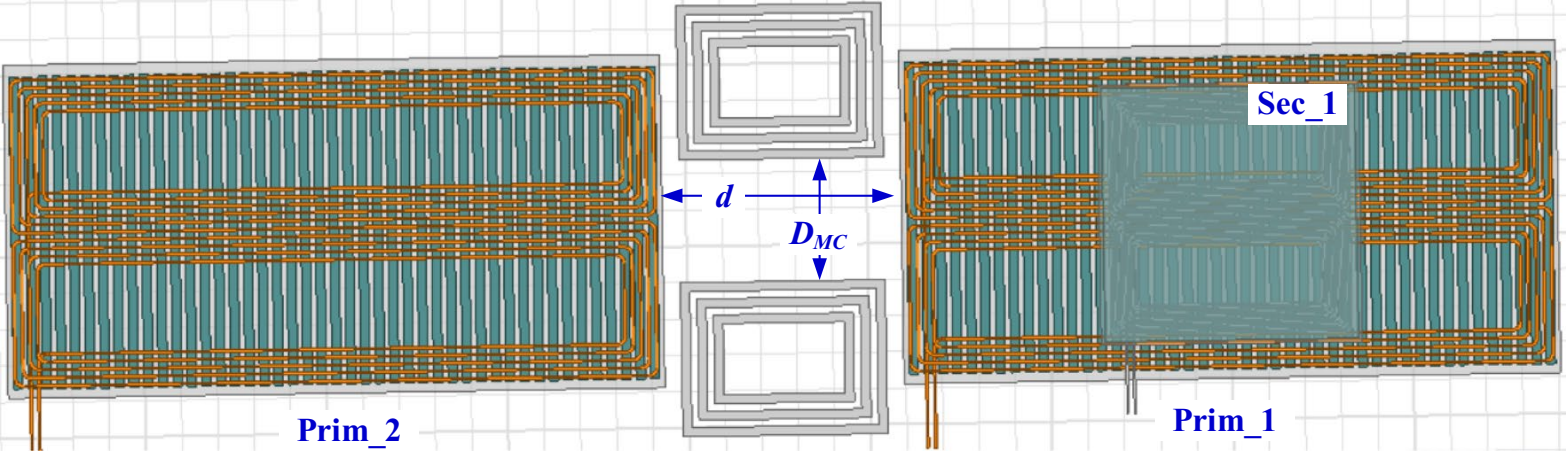
Top view of DTSR coupler with magnetizable concrete (MC) for DIPT system.

The greater the horizontal distance between the two transmitting coils (*d*), the lower the value of the total mutual inductance (*M*_*tot*_) between the transmitter and the receiver. To improve this value in large horizontal distances at the fixed operating frequency, it is proposed to insert magnetizable concrete (MC) in the area between the two transmitter coils. This MC bears the mechanical stresses and changing loads on the current urban roads, making the system more reliable and durable. An analysis based on several attempts was made to find the appropriate configuration of the MC that gives the highest value of *M*_*tot*_. In the beginning, bars of MC were used, which proved ineffective. Therefore, it was decided to create a configuration of MC in the form of DD, dividing between each D a distance of *D*_*MC*_, as shown in Fig. 18.

**Figure 19:**
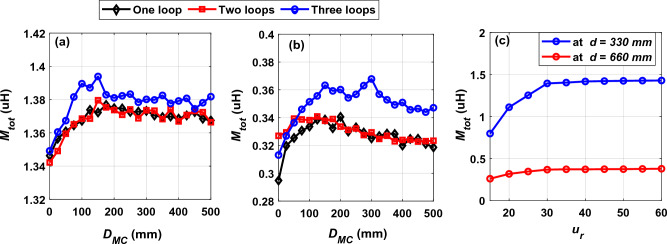
Performance of MC when introduced into the DIPT system, (**a**) *M*_*tot*_ vs. *D*_*MC*_ @ *d* = 330 mm, (**b**) *M*_*tot*_ vs. *D*_*MC*_ @ *d* = 660 mm, and (**c**) *M*_*tot*_ vs. *μ*_*r*_ @ *d* = 330 mm and *d* = 660 mm.

The first analysis was carried out on a DD core consisting of one rectangular loop, then the number of loops was increased to two, then three. The total mutual inductance *M*_*tot*_ between the transmitting and receiving coils was calculated when the distance* D*_*MC*_ changed from 0 to 500 mm in each case (one loop, two loops, and three loops) as shown in Fig. 19. Figure 19a shows that the highest value of the total mutual inductance *M*_*tot*_ is at the distance* D*_*MC*_ of 150 mm at the horizontal distance *d* of 330 mm, while Fig. 19b shows that the highest value of the total mutual inductance *M*_*tot*_ at the horizontal distance *d* of 660 mm when the *D*_*MC*_ distance is equal to 300 mm. In both horizontal distances (330 mm and 660 mm), the highest value of *M*_*tot*_ is obtained when the coil side of receiver is exactly opposite to the middle of each D on the transmitting side. The distance between the DD configuration loops was set at 5 mm. The DD configuration containing three rectangular loops was considered when calculating the total mutual inductance *M*_*tot*_, output power *P*_*o*_, and efficiency *η* along the driving path.

After achieving the appropriate configuration form of the MC that gives the highest *M*_*tot*_, the relative magnetic permeability (*μ*_*r*_) is tested to provide the highest mutual inductance. The *μ*_*r*_ was changed according to the range given in^26,29^, from 15 to 60 by step of 5. The values of the associated *M*_*tot*_ at the mid-distance between the two transmitting coils is extracted as illustrated in Fig. 19c. It can be seen that the relationship between the total mutual inductance and the relative magnetic permeability is non-linear where the value of *M*_*tot*_ increases with the increase of the *μ*_*r*_, then it reaches almost stability. *μ*_*r*_ values higher than 30 do not lead to a significant increase in the total mutual inductance. Therefore, the value of relative magnetic permeability in this study is considered to be 30.

**Figure 20:**
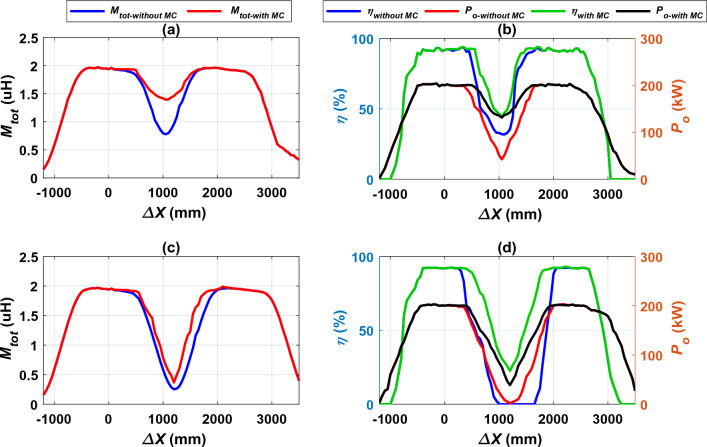
Performance of the DIPT system with magnetizable concrete (MC), (**a**) *M*_*tot*_ vs. *∆X* @ *d* = 330 mm, (**b**) *η*, *P*_*o*_ vs. *∆X* @ *d* = 330 mm, (**c**) *M*_*tot*_ vs. *∆X* @ *d* = 660 mm, and (**d**) *η*, *P*_*o*_ vs. *∆X* @ *d* = 660 mm.

The performance of the proposed DIPT charging system was studied in case of the presence of the MC in the horizontal distance between the two transmitting coils and compared with the basic design without the MC (at FF = 85 kHz). This comparison was conducted in terms of *M*_*tot*_, *P*_*o*_ and *η* as illustrated in Fig. 20. In case of *d* equal to 330 mm, the total mutual inductance *M*_*tot*_ with and without MC was compared as shown in Fig. 20a, where the value of *M*_*tot*_ was improved in the presence of MC by 44.15% at *∆X* of 1050 mm. In addition, studying the output power *P*_*o*_ and efficiency *η* along the driving path is presented as depicted in Fig. 20b. The *η* value was increased to 46.47%, while the *P*_*o*_ value was improved from 42.55 to 131.45 kW by percentage of 67.63%. The effect of magnetizable concrete (MC) on system performance is observed starting from *∆X* = 100 mm to *∆X* = 1750 mm along the driving direction. In Fig. 20c, the improvement in total mutual inductance *M*_*tot*_ was studied when the distance *d* is 660 mm, where it was found that the mutual inductance is improved by 31.13% when using the MC. Moreover, the *P*_*o*_ was improved from 1.91 to 38.87 kW by percentage of 97.41% and the *η* increased to 22.58% as illustrated in Fig. 20d. Furthermore, the effect of magnetizable concrete (MC) on system performance is observed starting from *∆X* = 100 mm to *∆X* = 2200 mm along the driving path.

The output power *P*_*o*_ and efficiency *η* values at the middle of distance *d* generated by the improvement process by using MC are summarized in Table 4.

**Table 4:** *P*_*o*_ and *η* values at the middle of distance *d* with the presence of the MC.

*d* (mm)	*∆X* (mm)	*M*_*tot-without MC*_ (μH)	*M*_*tot-with MC*_ (μH)	*P*_*o-without MC*_ (kW)	*P*_*o-with MC*_ (kW)	*%P*_*o-ripple*_	*η*_*-without MC*_ (%)	*η*_*-with MC*_ (%)	*%η*_*ripple*_
330	1050	0.778	1.394	42.55	131.45	67.63	31.94	46.47	31.26
660	1200	0.253	0.3675	1.91	73.87	97.41	0	22.58	22.58

From this comparison, it was concluded that the inclusion of magnetizable concrete in the magnetic design of the proposed charging system proved a good effectiveness and led to a significant improvement in the output power and efficiency in the case of *d* = 330 mm. On the contrary, in the case of the distance *d* is 660 mm. Despite the remarkable improvement, it is still not suitable for the proposed charging system. Therefore, the system can be studied by merging the two previous improvement methods together (magnetizable concrete with operating frequency control) in order to test the possibility of the best improvement of the system performance in case of large horizontal distances between the two transmitting coils, as will be presented in the next section.

### Improvement by MC with frequency control

**Figure 21:**
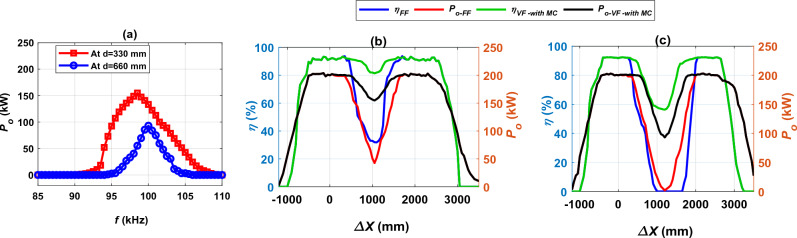
Performance of the DIPT system with magnetizable concrete (MC) and variable frequency (*VF*), (**a**) *P*_*o*_ vs. *f*, (**b**) *η*, *P*_*o*_ vs. *∆X* @ *d* = 330 mm, and (**c**) *η*, *P*_*o*_ vs. *∆X* @ *d* = 660 mm.

In this section the best possible improvement of output power and efficiency in case of large distances between the two transmitter coils has been conducted. To achieve this purpose, frequency control technique with magnetic concrete (MC) is applied for the proposed charging system. This technique has been applied in the area where the output power begins to fluctuate when moving along the driving path from *∆X* = 100 mm to *∆X* = 2200 mm. The variable frequency (*VF*) was changed from the fixed value (*FF*) 85–110 kHz with a step of 0.5 kHz. At each frequency the associated output power *P*_*o*_ value was calculated as illustrated in Fig. 21a. As the frequency increases from the fixed value, the *P*_*o*_ value increases to reach its highest value at the variable frequency (*VF*) of 98.5 kHz for a distance *d* of 330 mm and 100 kHz for a distance *d* of 660 mm.

The value of the output power *P*_*o*_ and transmission efficiency *η* at variable frequencies (98.5 kHz, and 100 kHz) was calculated and compared to its counterparts for the fixed frequency (85 kHz). At the distance *d* of 330 mm, a significant improvement occurred in the value of the *P*_*o*_, where it increased from 42.55 to 154.80 kW, with a percentage of 72.51%, as illustrated in Fig. 21b. Although the operating frequency increased by a small percentage of 13.7%, the *P*_*o*_ increased by a significant amount. In addition, there was a significant improvement by 60.76% in the transmission efficiency *η*, where its value reaches 81.40% at the same operating frequency. In case of distance *d* of 660 mm, there was an improvement in the *P*_*o*_ by 97.94%, as its value increased from 1.91 to 93.08 kW at the operating frequency of 100 kHz. In addition, the *η* was improved to reach 56.35% at the same frequency as depicted in Fig. 21c. The *P*_*o*_ and *η* values at the middle of distance *d* generated by the improvement process by controlling the operating frequency with the presence of the MC are summarized in Table 5.

**Table 5:** *P*_*o*_ and *η* values at the middle of distance *d* of the frequency control technique with the presence of the MC.

*d* (mm)	*∆X* (mm)	*M*_*tot-with MC*_ (μH)	*FF* (kHz)	*VF* (kHz)	*P*_*o-FF*_ (kW)	*P*_*o-VF-with MC*_ (kW)	*%P*_*o-ripple*_	*η*_*FF*_ (%)	*η*_*VF-with MC*_ (%)	*%η*_*ripple*_
330	1050	1.3938	85	98.5	42.55	154.80	72.51	31.94	81.40	60.76
660	1200	0.3678	85	100	1.91	93.08	97.94	0	56.34	56.34

### Technoeconomic analysis

**Figure 22:**
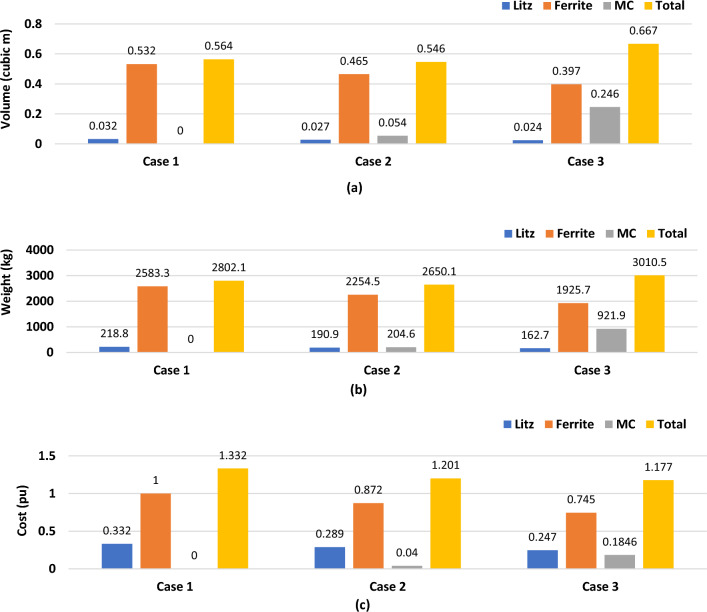
Comparison between volume, weight, and cost for different power pulsation improvement cases, (**a**) volume (cm^3^), (**b**) weight (kg), and (**c**) cost (pu).

Pulsating power improvement techniques can be divided into three cases. Case 1, when the distance between the two transmitter coils is small (*d* = 50 mm), then the power is improved by controlling the operating frequency. Case 2 and Case 3 in large distances at *d* = 300 mm and *d* = 660 mm respectively, then magnetizable concrete is used with operating frequency control to improve the pulsating power. These three cases are compared in terms of size, weight and cost for a 100-m driving track in order to reach the most cost-effective case as depicted in Fig. 22. According to the proposed transmitter coil dimensions listed in Table 1, the volume for each of the ferrite bars and Litz wires that make up the transmitter pad can be calculated. In addition, the possibility of calculating the weight of each of them using the information provided by the datasheet for ferrite^30^ and Litz wires^31^. It is also possible to calculate the volume and weight of the magnetizable concrete (MC) in cases 2 and 3 according to what was reported in^32^. Moreover, the cost of litz wire can be calculated given its length and the price per meter mentioned in^33^, while the cost of ferrite can be calculated by knowing the total volume along with the price of one ferrite bar^34^. The total cost of the system includes the cost of litz wires and the cost of ferrite bars in case 1, in addition to the cost of magnetized concrete in cases 2 and 3.

Case 2 records the lowest value for volume and weight if compared to cases 1 and 3 because it contains fewer transmitting coils with a small amount of magnetizable concrete (MC). On the contrary, in case 3, the volume and weight are greater because they contain a large amount of concrete in the space between the two transmitting coils. If the comparison is made from the economic point of view, although case 1 does not contain MC, it is the highest in cost due to the increase in the number of transmitting coils. While the cost of the case 3 is the smallest, despite the fact that it contains MC, due to the small number of transmitting coils. From this analysis, it can be concluded that the case 1 is suitable in terms of weight, volume, and less complicated in installation, but high in cost. Whereas in case 3 the cost is small, but it is larger in size, weight and more complex in installation.

## Conclusion and future work

This paper focuses on designing a magnetic coupler that suit with the in-motion charging. Therefore, it proposes a magnetic design for power of 200-kW with a transmission efficiency of 91.88% by using LCC-S compensation network. A design was made for each of the transmitter and receiver coils after obtaining the magnetic parameters from the mathematical model. The dimensions of the coils, ferrite and aluminum shielding were obtained, which give the magnetic parameters (*L*_*p*_, *L*_*s*_, *M*) that achieve the desired power. Also, the output power (*P*_*o*_) and transmission efficiency (*η*) were studied in the cases of perfect alignment and linear misalignment. In addition to that, the effect of inserting another transmitter coil at ground side was studied in term of *M*, *P*_*o*_, and *η.* To improve the output power during movement, the operating frequency was controlled in the case of small distances, then magnetizable concrete (MC) was inserted in the large distances, and then the system was tested in the presence of both together in the very large distances. These techniques proved effective in improving output power and efficiency, as power was improved by 45.15% and efficiency by 15.95% in the case of small distances (*d* = 50 mm). In the case of large distances, the improvement in power and efficiency were by 72.51% and 60.76% for *d* = 330 mm, and 97.94% and 56.34% for *d* = 660 mm respectively.

Depending on the analysis, findings, and conclusions presented in this manuscript, numerous research subjects emerge that can be good candidate for future research, that are listed below:

- Conduct extensive experimental testing for the proposed DD-DD in-motion design.
- Explore the compatibility and interoperability of the proposed transmitter with different receiver configurations such as circular, rectangular and solenoid.
- Investigate Electromagnetic fields (EMFs) issues for the proposed system.
- Study the possibility of designing a scalable system by adding two or more coils to the receiving side.

## Data Availability

The data used to support the findings of this study are available from the corresponding author upon request.
